# Fecal Microbiota Transplantation Effectively Cures a Patient With Severe Bleeding Immune Checkpoint Inhibitor-Associated Colitis and a Short Review

**DOI:** 10.3389/fonc.2022.913217

**Published:** 2022-06-10

**Authors:** Minmin Chen, Mengyuan Liu, Chenyan Li, Shiqiao Peng, Yiling Li, Xiuying Xu, Mingjun Sun, Xuren Sun

**Affiliations:** ^1^ Department of Gastroenterology, First Affiliated Hospital of China Medical University, Shenyang, China; ^2^ Department of Gastrointestinal Endoscopy, First Affiliated Hospital of China Medical University, Shenyang, China; ^3^ Department of Endocrinology and Metabolism, First Hospital of China Medical University, Shenyang, China

**Keywords:** colitis, fecal microbiota transplantation, immune checkpoint inhibitors, case report, immunotherapy

## Abstract

Immune checkpoint inhibitors (ICIs) have opened up a new way for tumor therapy but simultaneously led to the occurrence of immune-related adverse events. We report a case of successful treatment of PD-1 inhibitor-associated colitis with fecal microbiota transplantation (FMT). The patient was a palatal malignant melanoma who developed diarrhea and hematochezia accompanied by fever, gastrointestinal bleeding, and infection after the third treatment with PD-1 (Toripalimab). The patient received general treatment unsuccessful, corticosteroid therapy after initial success but rapid loss of response, and finally successful treatment after fecal microbiota transplantation.

## Introduction

Immune checkpoint inhibitors include anti-cytotoxic T lymphocyte antigen 4(CTLA-4), anti-programmed cell death 1(PD-1), and anti-programmed cell death ligand 1 (PD-L1) antibodies, which are monoclonal antibodies that block the inhibition of T cell function and enhance the cytotoxic activity of T cells, thereby improving anti-tumor immunity and being widely used in tumor immunotherapy (1, 2). However, the widespread application of immune checkpoint inhibitors has also led to an increase in immune-related adverse events (irAEs). These adverse events may occur throughout the body, and one of the most common adverse events is immune-related colitis (3).The principal clinical manifestations of ICI-associated colitis are abdominal pain, diarrhea, blood in the stool, nausea/vomiting, and fever. Currently, the treatment of immune-related colitis mainly depends on the severity of symptoms. Immune-related adverse events are classified into five grades according to the “Common Terminology Criteria for Adverse Events (CTCAE) 5.0”, defined as Grade 1: mild, Grade 2: moderate, Grade 3: severe or requiring hospitalization but not life-threatening, grade 4: life-threatening, and grade 5: death (4).It is generally recommended to perform symptomatic treatment and close observation for grade 1 irAEs. Grade 2 irAEs may require low to moderate doses of corticosteroids and a temporary cessation of immunotherapy. In high-grade (grades 3-4) irAEs, a high dose of corticosteroids is often required, along with immunosuppressives and discontinuation of immunotherapy (5, 6). However, both corticosteroids and anti-tumor necrosis factor α (TNF-α) drugs have significant side effects, long-term use may have risks of infection, diabetes, osteoporosis, etc. (7) Anti-integrin therapy (vedolizumab) seems safer and has also been reported to be effective in this disease, but it works slowly for severe patients (8, 9).Fecal microbiota transplantation (FMT), a method of putting feces from a healthy donor into the gastrointestinal tract of another patient to restore the balance of gut microbes, is widely used to treat refractory Clostrioides difficile infections (10, 11). In recent years, many studies have shown that fecal microbiota transplantation has a certain effect on inflammatory bowel disease (12, 13). Our patient was successfully treated with fecal microbiota transplantation after general therapies, glucocorticoid therapy, and Vedolizumab therapy failed.

## Case Report

**Figure 1:**
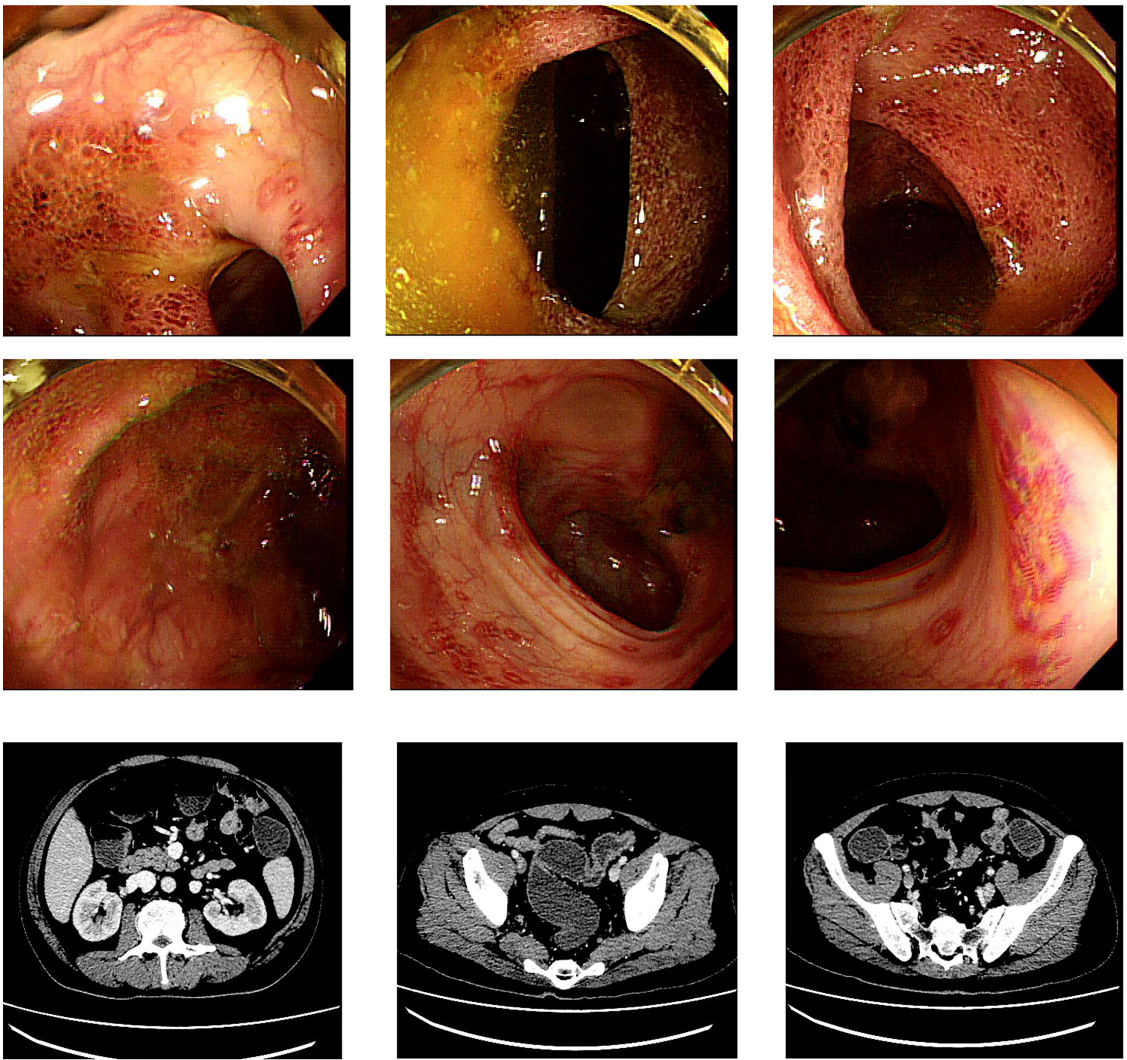
Colonoscopy and CT manifestations at diagnosis: Colonoscopy: extensive erythema, edema, fragility, loss of blood vessel texture, erosion and ulcers in the colorectal; CT: colorectal dilatation, effusion, visible air-fluid level.

**Figure 2:**
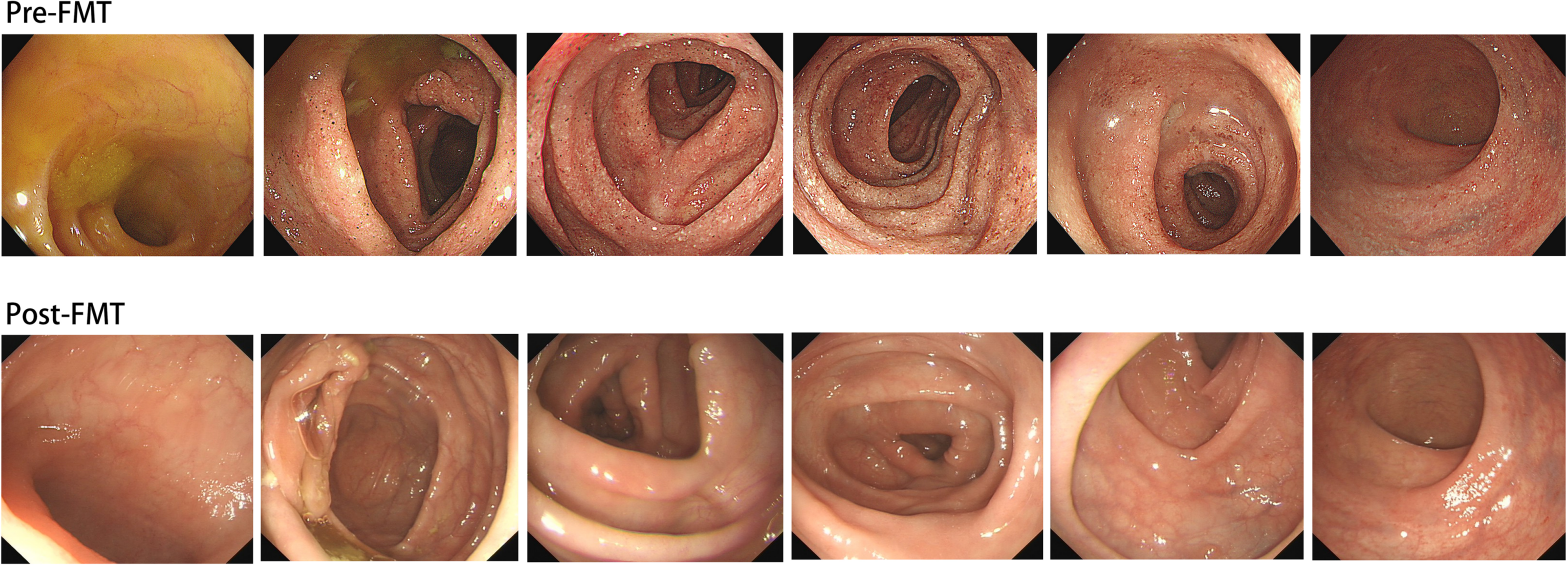
Colonoscopy manifestation before fecal microbiota transplantation but following glucocorticoid treatment and two doses of vedolizumab treatment; Colonoscopy evaluation after fecal microbiota transplantation suggests that the colon and rectum mucosa return to normal.

The patient was 49 years old, male, with malignant melanoma of the palate, and was treated with a PD-1 inhibitor (Toripalimab). The symptoms appeared after the third anti-PD-1 treatment. He went to the outpatient clinic for fever and CTCAE grade ≥ two diarrhea/colitis. Ineffective general treatments led him to hospitalization on the 10th day after onset. All infectious tests were negative, including cultures of common intestinal pathogens, PCR-based Epstein-Barr virus, cytomegalovirus, tuberculosis, and Clostridioides difficile. Colonoscopy showed widespread inflammation of the entire colon, which endoscopically resembled ulcerative colitis (**Figure 1**). CT examination suggested colon dilatation with effusion and visible gas-liquid level (**Figure 1**). He was treated with systemic corticosteroids (80mg IV daily, 1.2mg/kg), but the symptoms were not significantly relieved. After the 6th day of glucocorticoid treatment, the patient’s symptoms gradually worsened, manifesting as bloody stools without feces 10 to 20 times per day, abdominal pain, and high fever (**Table 1**). Continuous stool cultures showed positive results for pseudomonas aeruginosa. Following adequate antibiotic control, the symptoms remained stubborn and persistent, with CTCAE grade 3-4 diarrhea/colitis and severe intestinal microflora disorder (Cocci: bacillus =10-20:1). Despite two doses of anti-integrin therapy (vedolizumab), his symptoms still existed, and there was no significant improvement in colonoscopy evaluation (**Figure 2**). So, at the onset of 56 days, we performed three consecutive fecal microbiota transplantation treatments (40ml stool in 120ml of liquid each time, once every other day, administered *via* the feeding tube jejunum). The fecal microbiota donor came from a healthy child, who is a social volunteer after rigorous screening.

**Table 1:** Comparation of symptoms, signs and laboratory examination before and after treatment.

Symptoms and signs Laboratory examination	Before treatment	After treatment
**Diarrhea**	Up to 10-20 times per day	Strip and soft stool, about 1-2 days/time
**Blood in stool**	Bloody stool with little feces	without
**Fever**	Up to 40°C	without
**Abdominal pain and abdominal tenderness**	The left lower abdomen	without
**Leukocyte**	10.5*10^9/L	normal
**Hemoglobin**	69g/L	91g/L
**ALB**	27.8g/L	36.5 g/L
**CRP**	225.2mg/L	5.4mg/L
**Calprotectin (<200)**	>1800μg/g	<200
**Proportion of cocci and bacilli**	10:1	1:10
**Fecal leukocyte**	35/HP	negative

Following three times of FMT, the patient quickly achieved corticosteroid -free remission, and the symptoms completely disappeared. After the first FMT, the patient only experienced a mild fever and loose stools with a small amount of blood twice a night. After the second FMT, there were only a few bloodless yellow loose stools with mixed stools. For the third time, the stool was formed, soft and strip-shaped, defecating every 1-2 days. The patient was discharged from the hospital after FMT, and his disease course lasted 60 days.

**Figure 3:**
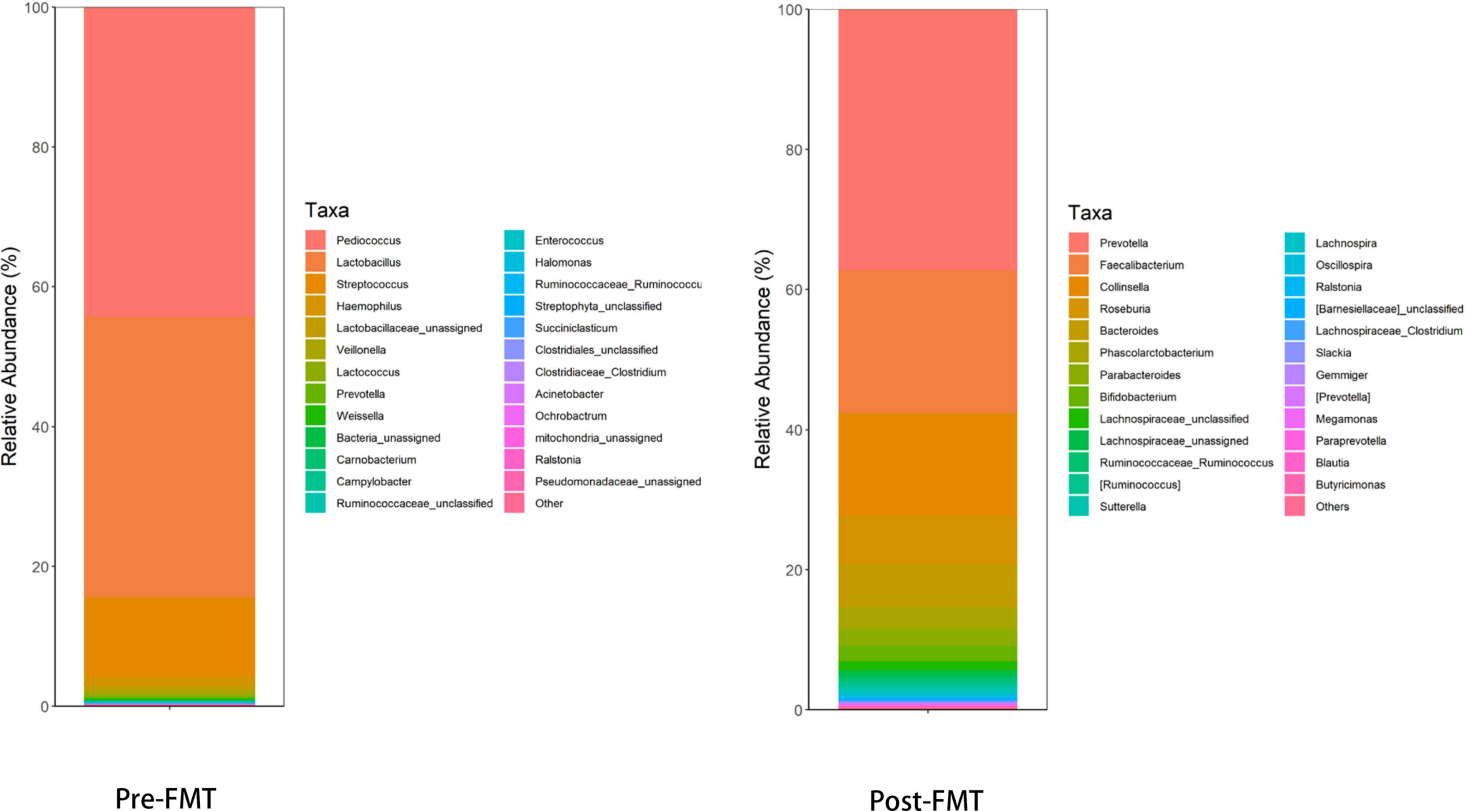
The level of the top 25 genera with higher abundance in the intestinal microflora before and after FMT and the total abundance of the remaining other bacterial genera: Pre-FMT, the abundance of various bacterial genera in the intestine was obviously abnormal, among which Pediococcus and Lactobacillus accounted for a higher proportion; Post-FMT, the structure of the intestinal microflora and the abundance of various bacterial genera in the intestine recovered significantly.

After applying glucocorticoid and two doses of anti-integrin therapy (vedolizumab), the patient’s colonoscopy evaluation showed no substantial improvement in colonic mucosal inflammation(**Figure 2**). Colonoscope assessment following FMT suggested that inflammation and erosion of the colon mucosa had subsided, and no signs of inflammation were left (**Figure 2**). Prior to and following FMT, we collected the patient’s stool samples for intestinal flora 16S gene sequencing. The results prior to FMT indicated that the diversity, structure, and abundance of microflora were obviously abnormal, and the detection of pathogenic bacteria also indicated the proportion of Campylobacter was increased (**Tables 2**、**3**, **Figures 3**、**4**). Intestinal flora 16S gene sequencing following FMT suggested that the diversity and the abundance of microflora were close to the normal range (**Tables 2**、**3**, **Figures 3**、**4**). No pathogenic bacteria were detected. The ratio of coccus to bacillus has also changed from 10:1 before FMT to 1:10, and CRP and Calprotectin have also returned to the normal range. Compared with the healthy FMT donor, the alpha diversity before FMT was variable, as measured by the Shannon index, and the gut microbiome diversity of patients became similar to the donor after treatment(**Figure 5**). With the relief of symptoms and increased food intake, the patient’s nutritional status also improved significantly (**Table 1**). Our patient started ICI treatment again six months after the cure. However, colitis recurred after the second anti-PD-1 treatment, manifesting as diarrhea, bloody stool 10-20 times per day, as before with abdominal pain and high fever. CT behaved similarly to previous episodes of colitis (**Figure 6**). Due to the economic reasons of the patient, we did not choose FMT this time, only glucocorticoids, vedolizumab, Mesalazine, and probiotics therapy were administered. The treatment proved effective, but the symptoms were slowly relieved. After 110 days of treatment, the patient continued to have mild enteritis, which manifested as occasional abdominal pain, and diarrhea 1-3 times a day with occasional small amounts of pus and blood. Colorectal mucosa still revealed active inflammation under endoscopy. Colonoscope assessment showed scattered patchy erosion in the entire colonic mucosa. The remainder of the intestinal mucosa is congested and edematous, and the texture of the blood vessels is blurred (**Figure 7**). According to the laboratory examination, moderate anemia, low albumin levels and high inflammatory markers continue to exist (**Table 4**). Comparing the results of the two treatments, it appeared that glucocorticoid therapy was less effective than fecal microbiota transplantation, at least for this patient. The comparison of the therapeutic effect between FMT and glucocorticoid therapy is shown in the table below (**Table 4**). At present, the patient has a recurrence of the tumor in the pelvis. Due to the recurrence of colitis, he did not receive immunotherapy but only received chemotherapy.

**Table 2:** Genetic testing of intestinal flora prior to and following FMT.

Genetic testing of intestinal flora	Pre-FMT	Post-FMT
**Microflora diversity (5.076-6.211)**	2.305	4.617
**Detection of pathogenic bacteria**	Campylobacter (0.077%)	Undetected
**Microflora structure**	The abundance of various bacterial genera in the intestine was obviously abnormal, among which Pediococcus accounted for 44.351% (higher), and Lactobacillus accounted for 40.068% (higher)	The abundance of Collinsella was high compared with the reference range. The abundance of Gemmiger was low compared with the reference range.

**Table 3:** Change of abundance of various bacterial genera prior to and following FMT.

Bacteria name	Normal range	Pre-FMT	Post-FMT
**Bacteroides**	9.921-32.231	0.003	6.144
**Prevotella**	0.000-16.547	0.281	37.274
**Faecalibacterium**	3.394-14.854	0.0008	20.359
**Roseburia**	0.441-7.535	0.000	6.884
**Collinsella**	0.000-0.352	0.000	14.653
**Ruminococcus**	0.626-1.158	0.040	0.820
**Parabacteroides**	0.994-1.034	0.000	2.477
**Phascolarctobacterium**	0.036-0.064	0.000	3.119
**Bifidobacterium**	0.456-1.468	0.015	2.181
**Blautia**	0.057-0.679	0.000	0.092
**Butyricimonas**	0.104-0.160	0.000	0.083
**Streptococcus**	0.049-0.507	11.322	0.000
**Lactobacillus**	0.000-0.589	40.068	0.068
**Gemmiger**	2.915-5.353	0.003	0.145
**Clostridium XIVa**	0.900-4.260	0.000	0.210

**Figure 4:**
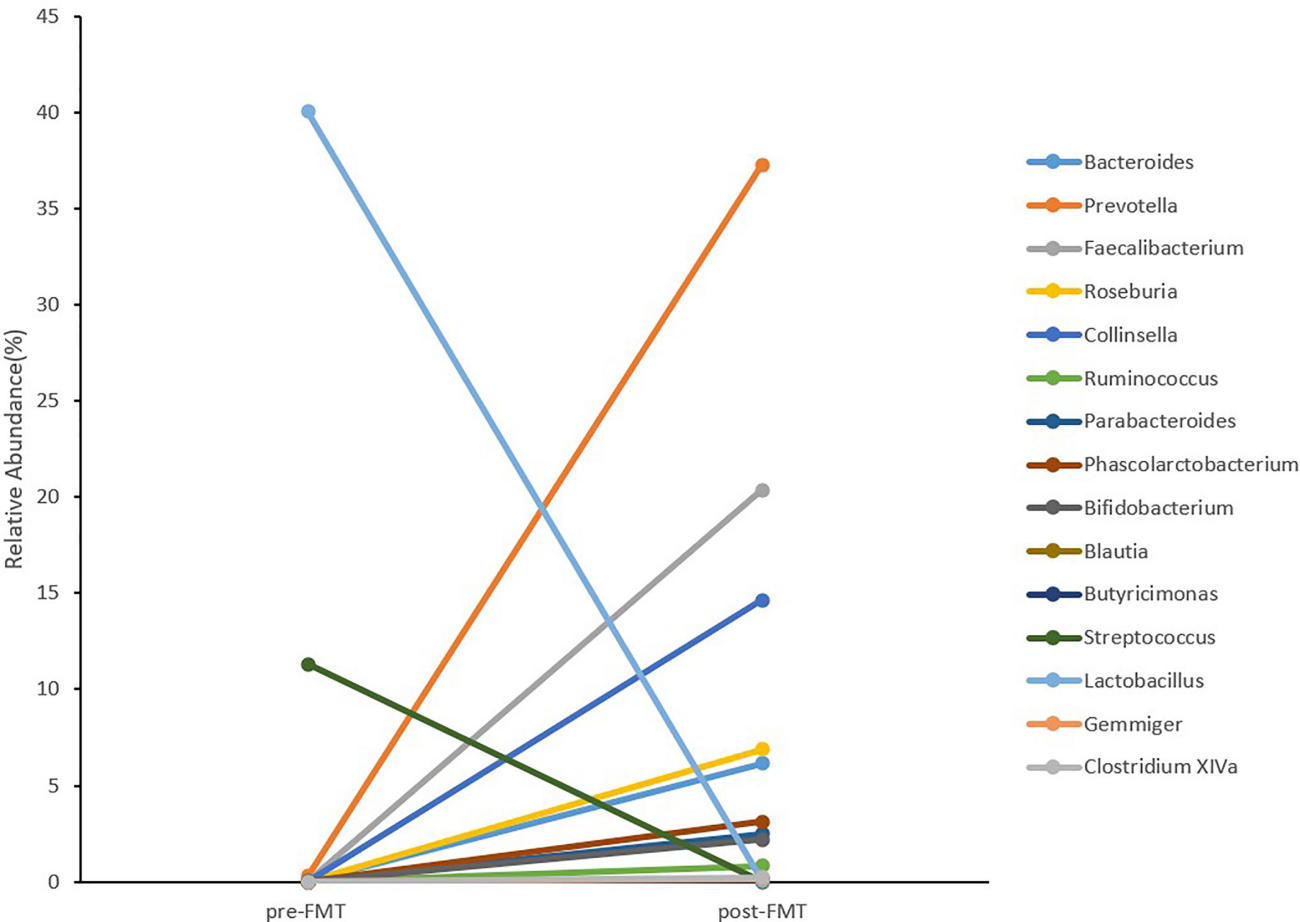
The top 15 genera abundance with wide variations before and after FMT.

**Figure 5:**
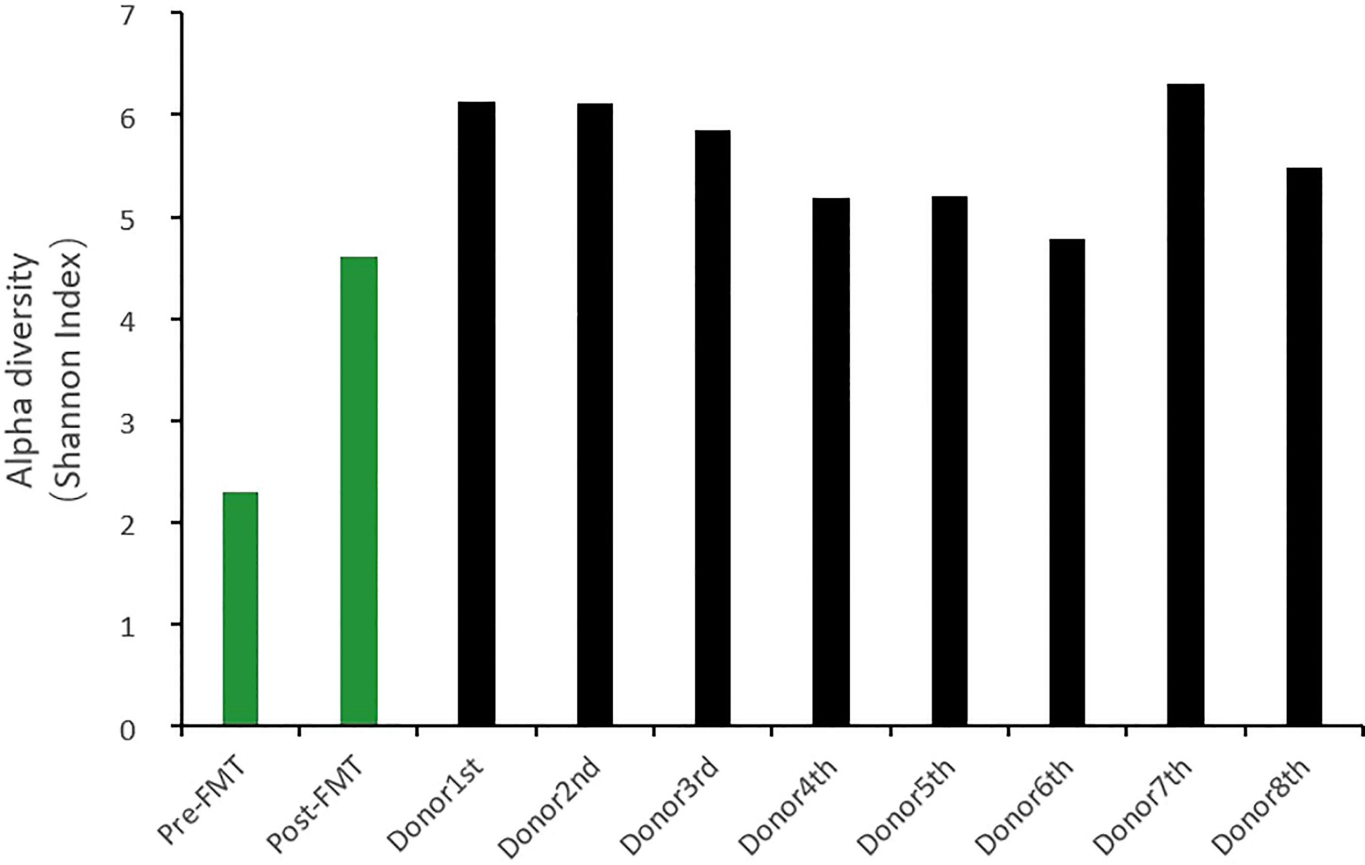
Donor1-8: 16S sequencing of the microflora abundance of the donor regularly monitored one year before donation (about every one and a half months or so) within 1 year. The data of the stool at the 8th time is the closest to the stool received by the patient. Compared with the healthy FMT donor, the alpha diversity before treatment was different, as measured by the Shannon index, and the gut microbiota diversity of patients became similar to the donor after treatment.

**Figure 6:**
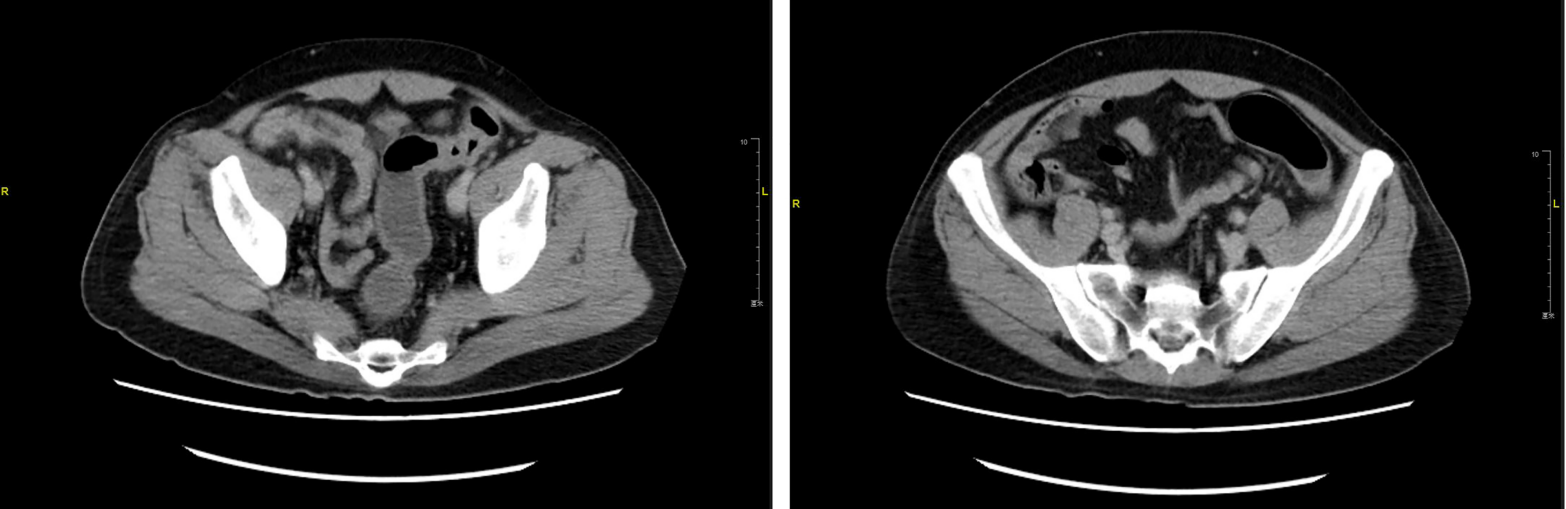
CT manifestation after recurrence.

**Figure 7:**
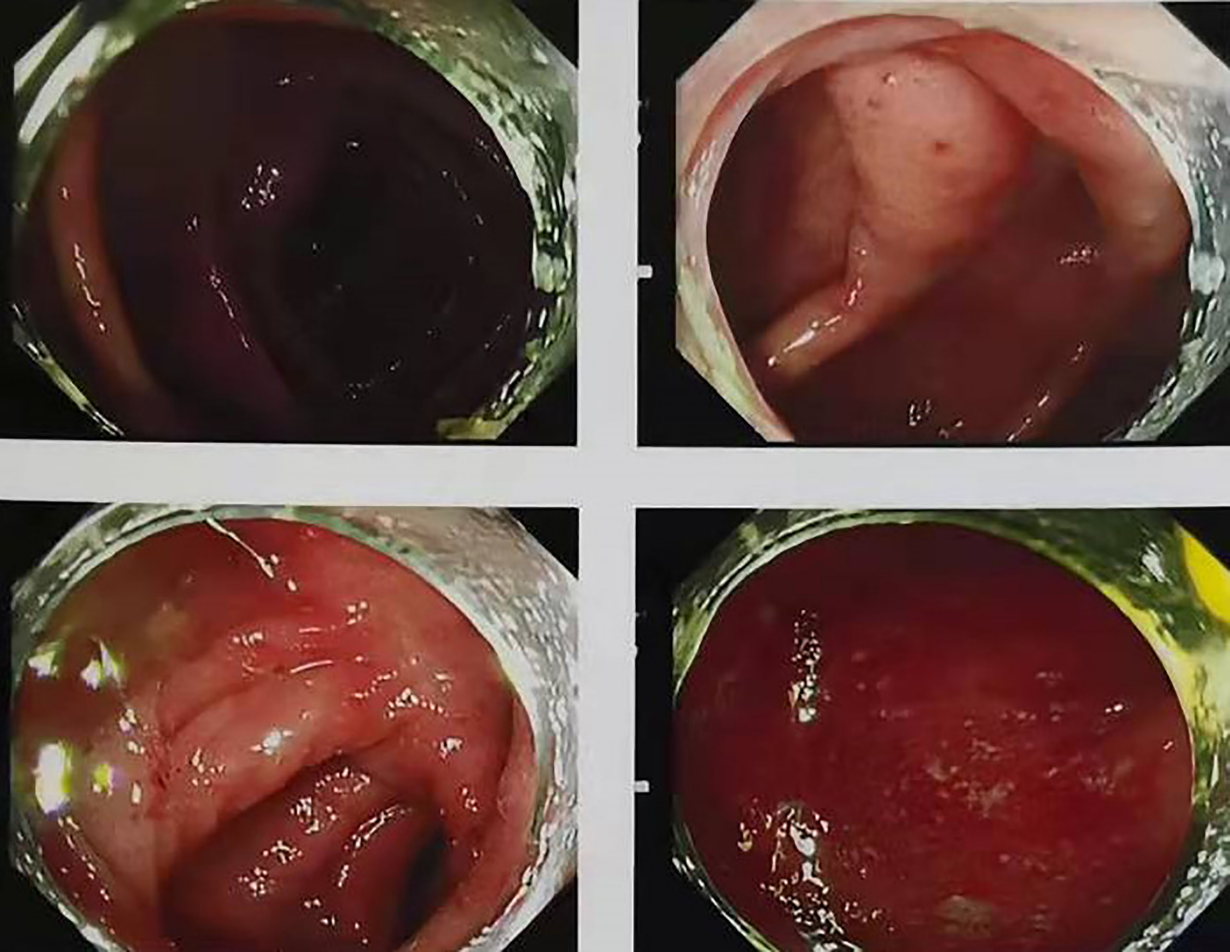
Colonoscopy manifestations following glucocorticoid therapy: scattered and patchy erosions can be seen in the entire colonic mucosa, the rest of the intestinal mucosa is congested and edematous, and the texture of the blood vessels is blurred.

**Table 4:** The comparison of the therapeutic effect between FMT and glucocorticoid therapy.

Therapeutic effects	Following FMT	Following glucocorticoid therapy
**Diarrhea**	strip and soft stool, about 1-2 days/time	diarrhea 1-3 times a day with occasional small amounts of pus and blood
**Abdominal pain**	Without	occasional
**Fever**	Without	Without
**CRP**	3.6mg/L	142.5mg/L
**Calprotectin (<200)**	<200μg/g	>1800μg/g
**Proportion of cocci and bacilli** **Hemoglobin** **ALB**	1:10 Increase significantly 36.5 g/L	1:5 Decrease,92 g/L 33.0 g/L
**Fecal leukocyte**	Negative	positive
**Fecal occult blood**	Negative	positive
**Treatment time**	7days	>110days
**Colonoscope manifestation**	The colon and rectum mucosa return to normal.	Scattered and patchy erosions can be seen in the entire colonic mucosa, the rest of the intestinal mucosa is congested and edematous, and the texture of the blood vessels is blurred.

## Discussion

Colitis is one of the most common adverse reactions associated with immune checkpoint inhibitors. IrAEs usually occur three months after the onset of ICI, but can also occur a day after the first injection, or even years after the onset of ICI (5). With different immune checkpoint inhibitors, the probability of adverse events is also different. Based on a meta-analysis, the incidence of anti-CTLA-4 antibody-associated irAEs was estimated to be 72%, while high-grade irAEs were 24%. In patients treated with anti-PD-1 and anti-PD-L1 antibodies, the overall incidence of irAEs was similar at 74%, but high-grade irAEs were fewer, at 14% (5). Combination therapy with anti-CTLA-4 and anti-PD-1 antibodies results in the highest frequency of irAEs, 88%, and 41% of high-grade irAEs (14). This indicates that the combined use of multiple ICIs can significantly increase the incidence and severity of irAEs. In contrast to patients treated with anti-PD-1 and anti-PD-L1, anti-CTLA-4 therapy is the most likely to lead to immune-related colitis (3).

According to the “Common Terminology Criteria for Adverse Events (CTCAE) 5.0”, immune-related adverse events are classified into four grades, defined as Grade 1: mild, Grade 2: moderate, Grade 3: severe or requiring hospitalization but not life-threatening, grade 4: life-threatening, and grade 5: death. Clinical manifestations of immune-related colitis usually involve the lower digestive tract, with abdominal pain, diarrhea, and hematochezia. After the infection is ruled out, endoscopy and biopsy are the primary diagnostic methods for colitis. The microscopic manifestations of immune-related colitis may be normal, maybe ulcers, or non-ulcerous inflammatory manifestations, such as erythema, edema, erosion, exudation, loss of vascular pattern, and bleeding (4). However, the severity of clinical symptoms may not be consistent with the endoscopic manifestations. Even if the endoscopic mucosal manifestations are normal, the possibility of colitis cannot be excluded. Due to the similar microscopic features between inflammatory bowel diseases and immune-associated colitis, mucosal tissue biopsy is often required. The histological features of immune-associated colitis can generally be divided into acute inflammation and chronic inflammation. Acute inflammation is characterized by neutrophilic or eosinophilic infiltration, cryptitis, crypt abscess, and apoptosis. Chronic inflammation is characterized by basal lymphocyte infiltration, cryptic architecture distortion, and Paneth cell metaplasia (15). In contrast, inflammatory bowel disease usually manifests as granulomas or chronic structural changes such as shortening of crypts and basal lymphoplasmacytosis (16). According to the American Society of Clinical Oncology Clinical Practice Guidelines, corticosteroids are currently the most frequently used first-line treatment for grade 2 or higher adverse events. For corticosteroid-refractory colitis, immunosuppressant therapy can be applied. Among them, infliximab has been widely used, and corticosteroid should continue to be used simultaneously. Infliximab is an anti-TNF-α antibody that has a significant impact on colitis (17). Recently, Vedolizumab, an intestinal-targeting α4β7 antibody, is considered an effective and safe therapy for patients with ICI colitis due to its mechanism of action being limited to lymphocyte transport in the gastrointestinal tract (8). It works by blocking the interaction between the α4β7 integrin found on the surface of T cells and the mucosal vascular address cell adhesion molecule 1 (MAdCAM-1) expressed on the surface of the venule endothelium in the venules and related lymphoid tissues in the gastrointestinal tract, thereby preventing leukocytes from binding to the endothelium surface and infiltrating into the affected tissues, thus achieving selective gastrointestinal immunosuppression (9).

Healthy people have a wide range of microorganisms in their intestines. The healthy gut microbiota is a diverse, stable, resistant, and resilient microbial ecosystem, and their composition plays a significant role in regulating their immunity. Studies have recently found that Clostrioides difficile-associated colitis and inflammatory bowel disease can be successfully treated with fecal microbiota transplantation. Following treatment, the intestinal microflora of patients showed distinct characteristics (18, 19) Similarly in a case series, after FMT, the increase of the abundance of Blautia and Bifidobacterium species contributes to the inflammation remission. It provides a potential and novel treatment of ICI colitis reliant on the modulation of gut microbiome (20). Indeed, according to a meta-analysis, FMT was considered to have a positive effect on the gut microbiome and leads to increased immunological responses while reducing irAEs in several studies. Therefore, in ICI colitis, the use of gut-microbiome altering drugs, such as antibiotics, should be carefully considered (21).Moreover, several clinical studies have indicated that the diversity of gut microbiota is associated with the outcome of cancer immunotherapy, and that manipulating gut microbiota improves the response to ICI treatment in turn enhancing the efficacy of anticancer immunotherapy (22). Gopalakrishnan has observed that in melanoma patients, different responses to immunotherapy were associated with differences in gut microbiota. Patients with abundant Faecalibacterium responded to anti-PD-1 therapy well. After FMT from a human patient who had responded well to anti−PD−1 therapy, these differences will disappear. This may be related to increased antigen presentation (23).Fecal microbiota transplantation directly regulates the balance of gut microbes by placing feces from a healthy donor into the patient’s gastrointestinal tract. In most cases, doctors put a small amount of liquefied and filtered feces into the colon through a colonoscopy. There are strict inclusion and exclusion criteria for stool donors and a comprehensive medical evaluation is required to avoid infections and minimize risks (10, 11). In the future, FMT could be a new first-line therapy if it proves more beneficial than current first-line therapies.

After systematic treatment, most irAEs disappear after stopping ICI therapy. Our patient started ICI treatment again after remission but continued to suffer from colitis. At present, the safety of restarting immune checkpoint inhibitor therapy cannot be predicted. In a cohort study of 180 people, it was found that the probability of recurrence of irAEs was 38.9%, and the same ICI treatment regimen may lead to a lower recurrence rate. The recurrence rate of gastrointestinal-related adverse events is higher than other adverse events (24). Another study found that restarting ICI therapy was more likely to result in fatal immune-related adverse events than initial ICI therapy (5). Initial immunosuppressive therapy and long duration of ICI symptoms were proved to be associated with a higher risk of ICI recurrence (25). Current oncology guidelines recommend permanent discontinuation of ICIS therapy for patients with the most severe irAEs (grade 4). For the second or third level, anti-CTLA-4 agents should be considered permanently cease. Anti-PD-1 is recommended to be permanently discontinued for certain grade 2 irAEs, such as myocarditis. If the patient can recover to grade 1 or lower, anti-PD-1 and anti-PD-L1 drugs can be restarted (17). In the future, prospective studies are required to assess the risk factors affecting the prognosis of patients after ICI restart.

With the continuous development of cancer immunotherapy, the application of immune checkpoint inhibitors will become more widespread, and the incidence of ICI-related colitis is also on the rise. Preventing and early prediction is one of the most worth exploring problems at present. A retrospective analysis of 213 patients found that vitamin D intake during ICI therapy significantly reduced the incidence of colitis in patients with advanced melanoma. This result indicates that prophylactic use of vitamin D supplements may associated with reduced risk for ICI-colitis (26). Previously, vitamin D has been a protective factor for inflammatory bowel disease (27). The composition of gut microbiota can also influence the development of inflammatory diseases. Bacteroides limit inflammation by stimulating the differentiation of T regulatory cells. According to reports, patients with a higher abundance of Bacteroides have a lower risk of colitis when receiving anti-ICI therapy (18, 28). Cytokines play an important role in promoting immune homeostasis. More and more evidence has showed that irAEs are related to specific cytokines, which promote the development of inflammation and may have predictive value for irAEs (29). In addition, fecal calprotectin, lactoferrin, and C-reactive protein (CRP) are currently known inflammatory markers, and early monitoring can help identify dynamic changes in inflammation (30). By identifying biomarkers that predict the risk of colitis, early diagnosis and treatment may be possible. Unfortunately, there are relatively few predictive markers that can predict ICI colitis.

Fecal microbiota transplantation provides a new idea for refractory colitis. Through controlling intestinal infection, improving gut microbiota, and controlling inflammation, can shorten the time of corticosteroid -free remission and enhance the therapeutic effect of ICI colitis. We performed fecal microbiota transplantation on patients at the later stage of the disease course. It is unclear whether early acceptance of fecal microbiota transplantation will make a difference in the prognosis of patients. In the future, more attention needs to be paid to how to predict the occurrence of ICI colitis early and whether it is possible to identify the risk factors for immune-related adverse events before ICI treatment, the probability of recurrence of adverse events after ICI treatment is restarted, and whether it can be prevented in advance to relieve symptoms and to reduce recurrence risk.

## Author Contributions

MC wrote the first draft of the manuscript. XS contributed to conception and reviewed the manuscript. ML, CL, and SP contributed to data curation and visualization. YL, MS, and XX analyzed the data and participated in the coordination of the study. All authors contributed to manuscript revision, read, and approved the submitted version.

## Conflict of Interest

The authors declare that the research was conducted in the absence of any commercial or financial relationships that could be construed as a potential conflict of interest.

## Publisher’s Note

All claims expressed in this article are solely those of the authors and do not necessarily represent those of their affiliated organizations, or those of the publisher, the editors and the reviewers. Any product that may be evaluated in this article, or claim that may be made by its manufacturer, is not guaranteed or endorsed by the publisher.
